# RETRACTION: Molecular Changes in Diabetic Wound Healing following Administration of Vitamin D and Ginger Supplements: Biochemical and Molecular Experimental Study

**DOI:** 10.1155/ecam/9865841

**Published:** 2026-04-09

**Authors:** Evidence-Based Complementary and Alternative Medicine

RETRACTION: H. A. Al‐Rawaf, S. A. Gabr, and A. H. Alghadir, “Molecular Changes in Diabetic Wound Healing following Administration of Vitamin D and Ginger Supplements: Biochemical and Molecular Experimental Study,” *Evidence-Based Complementary and Alternative Medicine*, 2019, 4352470, https://doi.org/10.1155/2019/4352470.

The above article, published online on 21 July 2019 in Wiley Online Library (https://wileyonlinelibrary.com), has been retracted by John Wiley & Sons Ltd. The retraction has been agreed due to concerns raised with Figure 3, initially raised on PubPeer [1]. On further investigation, additional concerns were identified:

• A large majority of the images shown in Figure 3 are identical or highly similar to images shown in Figure 2 of [2] (in some cases, after minor stretching and/or re‐sizing).
• The Diabetic Day 15 panel in Figure 3 appears similar to the VD treatment group Day 9 panel in Figure 6a of [3].
• The Diabetic Day 6 panel appears similar to the Diabetic group Day 6 panel in Figure 6a of [3].
• The GF Day 15 panel appears similar to the fourth control panel in Figure 4a of [4].
• The Vitamin D Days 6 and 9 panels appear similar within Figure 3.
• The Vitamin D Day 9 panel and GF + Vita D Day 6 panel appear similar within Figure 3.
• The Control Day 9 panel appears similar to the GF Day 3 panel within Figure 3.

After assessment of the concerns and author’s response by a subject matter expert, additional concerns with the methodology and qPCRpcr data were identified and retraction was recommended. As such, the article is retracted.

The authors disagree with the retraction.
